# The effect of COVID-19 pandemic on respiratory therapy students’ clinical practice: a cross-sectional study

**DOI:** 10.1186/s12909-023-04340-y

**Published:** 2023-05-26

**Authors:** Aseel Jamal Baoum, Elaf Asaad Hadidi, Renad Fahad Alharbi, Muhammad Anwar Khan, Alqassem Y. Hakami

**Affiliations:** 1 College of Applied Medical Sciences, King Saud bin Abdulaziz University for Health Sciences, Jeddah, Saudi Arabia; 2 College of Medicine, King Saud bin Abdulaziz University for Health Sciences, Jeddah, Saudi Arabia; 3 King Abdullah International Medical Research Center, Jeddah, Saudi Arabia

**Keywords:** COVID-19, Students, Universities, Clinical practice, Medical Education

## Abstract

**Background:**

Due to the 2019 Coronavirus disease (COVID-19) precaution, educational systems and learners’ practices from all specialties have been negatively affected, especially university students. COVID-19 has a massive effect on the practice of allied health students. The students’ hospital exposure has been severely affected by the cancelation of the clinical practice. This study aims to investigate the effect of COVID-19 pandemic on the clinical practice of respiratory therapy students in different universities around Jeddah, Saudi Arabia.

**Methods:**

Analytical cross-sectional online questionnaire was distributed from August 2021 to November 2021 to respiratory therapy students. The study’s sampling technique was non-probability consecutive, and the calculated sample size was 183 participants. The survey contained questions to identify the clinical exposure of the participants. The participants included RT students in their clinical training years from King Saud bin Abdulaziz University for Health Sciences, King Abdulaziz University, and Batterjee Medical College in Jeddah. The survey evaluated the effects of the pandemic on students’ clinical practice, confidence and clinical preparation, and education.

**Results:**

A total of 187 respiratory therapy students completed the questionnaire. The results revealed that 145 (77.5%) of respiratory therapy students agreed that the pandemic had disrupted their clinical practice. The percentage of respiratory therapy students who felt that they were less confident and less prepared for the next academic year due to practical session cancellation was 141 (75.4%). Out of the total students, 135 (72.2%) students reported difficulty in connecting the clinical and theoretical part because of the pandemic.

**Conclusion:**

The majority of respiratory therapy students from the three universities similarly reported that the pandemic disrupted their practice and interfered with their ability to connect between clinical and theoretical part. Moreover, it had affected their confidence and preparedness for the next year.

## Introduction

Respiratory therapy is the health care specialty that focuses on cardiopulmonary system function and its disorders. A respiratory therapist (RT) is a professional healthcare practitioner that concerns with the prevention, diagnosis, and management of all cardiopulmonary dysfunctions [1]. Respiratory therapists have various options when it comes to the site of work; however, about 75% of all RTs are utilized in the hospitals. RTs cover a broad range of patients from neonates to elderlies [1–3]. Before the start of the Novel Coronavirus disease (COVID-19) pandemic crisis, the majority of the population had little knowledge about respiratory therapists. Nowadays, respiratory therapists have an integral role in managing patients with COVID-19 which allowed them to be well-known to the population [4–7].

COVID-19 has affected the quality of life in many different ways. It has affected educational systems worldwide and forced all educational institutions to shift from conventional education to online and distance learning to reduce its spread among students [8]. The pandemic has a massive effect on learners and their practices from all specialties, especially university students [9, 10]. COVID-19 has become a challenge for their educational experiences and all students have significant concerns about missing out on their practical part of learning, which has a crucial role in understanding their curriculum [11, 12].

Allied health students’ practice has been negatively affected by this pandemic [13]. Harries et al. study in 2020 has revealed that the COVID-19 pandemic has affected medical students’ education and clinical practice [14]. Moreover, Ferrara et al. study in 2020 has demonstrated the effects of the COVID-19 pandemic on reshaping ophthalmology trainees training [15]. To the best of our knowledge, no studies have investigated the effect of the COVID-19 pandemic on respiratory therapy students’ clinical practice.

The curriculum of respiratory therapy includes clinical practice sessions in the third, fourth, and fifth years of their study journey. In addition, it focuses on mechanical ventilation management for critical condition and several skills required for RT. Therefore, their clinical rotations were conducted mainly in intensive care unit (ICU) in which the training occurs on critical patients under supervision of registered RT staff. The curriculum of RT highly depends on connecting the knowledge they have studied theoretically with the skills they have trained clinically. With the pandemic precaution, it became a challenge for RT students to continue their normal educational journey.The students’ hospital exposure was less and most of them had canceled clinical practice which affected their ability to connect between clinical and theoretical part.

According to the previous mentioned data, the study will examine whether the COVID-19 pandemic and concurrent virtual settings affect the respiratory therapy students’ clinical practice or not. This study aimed to investigate the effect of COVID-19 pandemic on respiratory therapy students’ clinical practice in Jeddah. Moreover, it examined the effects on respiratory therapy students’ confidence and preparation for clinical practice in addition to the effects on education due to the lack of connection between clinical and theoretical part.

## Methodology

### Study Design and setting

Analytical cross-sectional questionnaire helped us to examine the effects of the exposure in our study population. The study population included students from third, fourth, and fifth year of both genders studying respiratory therapy during COVID-19 pandemic. The targeted institutes were King Saud bin Abdulaziz University for Health Sciences (KSAUHS), King Abdulaziz University (KAU), and Batterjee Medical College (BMC) in Jeddah, Saudi Arabia. The exclusion was respiratory therapy students who dropped before the end of the semester. The time frame in which the study conducted was from August 2021 to November 2021.

### Participants and sample size

The sample size was calculated by using the Raosoft® software by the website www.raosoft.com. KSAUHS has a total of 150 respiratory therapy students, KAU has a total of 81 respiratory therapy students, and BMC has a total of 114 respiratory therapy students. The total of respiratory therapy students in the third, fourth, and fifth year of the three universities are 345 students. The required sample size was estimated at the 95% confidence level with 50% response distribution and a margin of error of ± 5%. The required minimum sample size was determined to be 183 participants. The study’s sampling technique was non-probability consecutive as we included whole population to get representative response.

### Variables and questionnaire

The studied variables included independent variables which were gender, academic year, and university, and dependent variables were the effects on practice and the effects on education. The data collection method of the research was online questionnaire distributed among RT students. The survey contained questions to identify the clinical exposure of the participants. The participants included RT students in their clinical training. An informed consent form was included on the first page of the questionnaire in addition to three questions sections. The first section included demographics information, such as gender, academic year, and university. In the second section, the questions examined the effects on the clinical practice, and the third section evaluated the effects on education. Responders were asked to answer on a likert scale (1 = strongly disagree, 2 = disagree, 3 = somewhat disagree, 4 = neither disagree nor agree, 5 = somewhat agree, 6 = agree, and 7 = strongly agree) which was used in the second and third sections. The questionnaire was adopted from a previous report that focused on evaluating the effects of the COVID-19 pandemic on medical students, and it was already valid and reliable [14]. Items of the questionnaire were evaluated for clarity of wording and consistency to assess perceptions of the following domains: effect on education and effect on practice and revised until consensus was reached. Permission from the study’s authors was acquired to utilize the questionnaire through email communications.

### Statistical methods

Data was entered using Microsoft Excel data sheet and was coded and prepared for statistical analysis. It was analyzed using Statistical Package for Social Sciences (SPSS) version 20.0. Respondent characteristics and key responses were summarized as counts and percent. Positive responses (somewhat agree, agree, or strongly agree) were combined into an agreement percentage in addition to negative responses (somewhat disagree, disagree, or strongly disagree) that were combined into a disagreement percentage. Chi-squared test was used to assess the association between categorical variables like gender, academic year, and university with different domains. P-value < 0.05 was taken as significant.

## Results

### Demographics

A total of 187 students completed the questionnaire. The number of female responses was 96 (51.3%). KSAUHS had the highest responses of 71 (38%) followed by KAU with responses of 62 (33.2%). Furthermore, the third year students’ number was 75 (40.1%) and fourth year students’ number was 67 (35.8%). Rest of demographic characteristics of the students are illustrated in Table 1.

**Table 1:** Demographics of the participants

	( n = 187) n (%)	
Gender:		
Male	91 (48.7)	
Female	96 (51.3)	
University:		
King Saud bin Abdulaziz University for Health Sciences - Jeddah	71 (38.0)	
King Abdulaziz University	62 (33.2)	
Batterjee Medical College	54 (28.9)	
Academic Year:		
Third	75 (40.1)	
Fourth	67 (35.8)	
Fifth (Intern)	45 (24.1)	

### Effects on practice and education

**Fig. 1:**
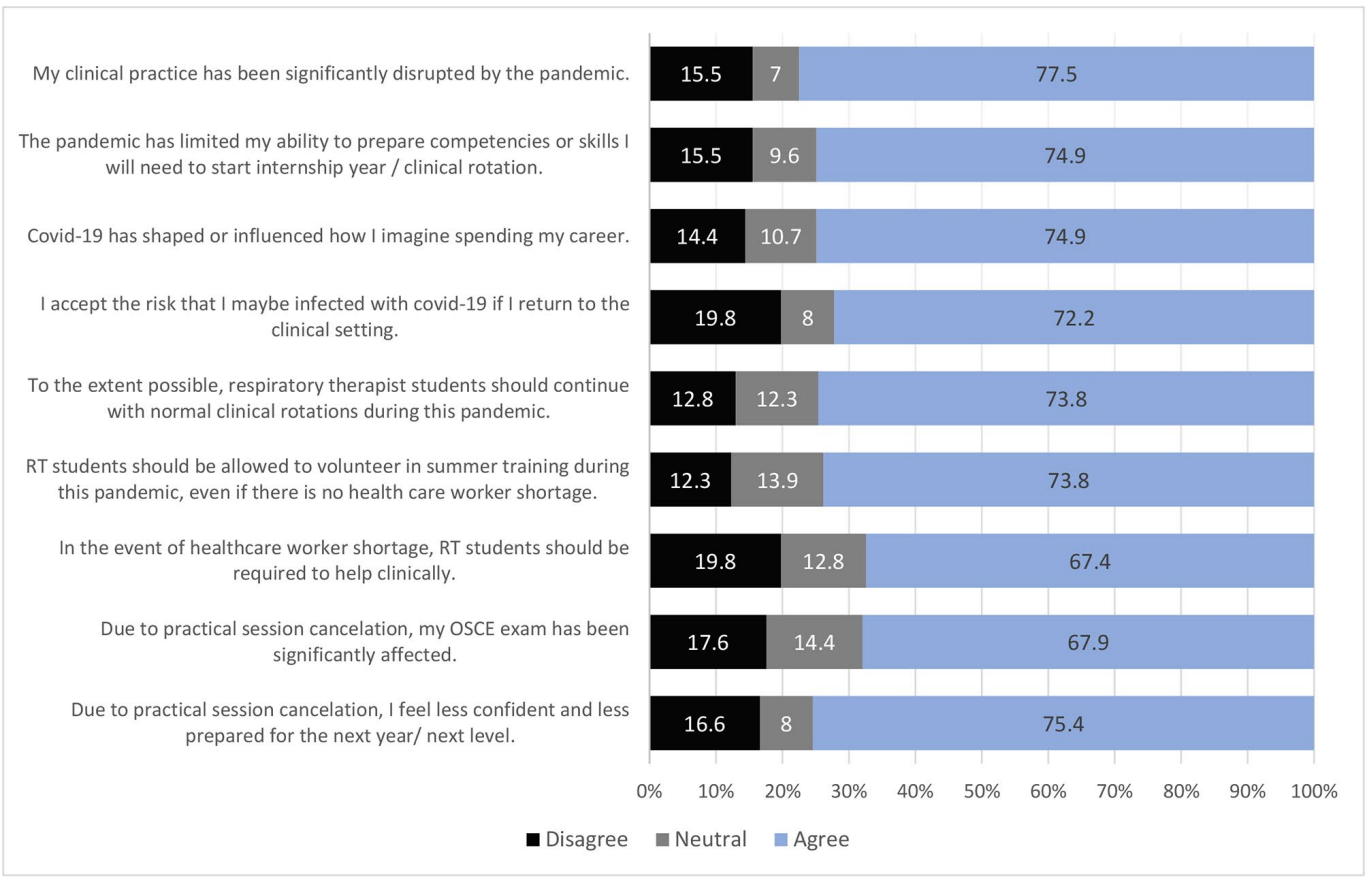
Examination of the effects on practice

**Fig. 2:**
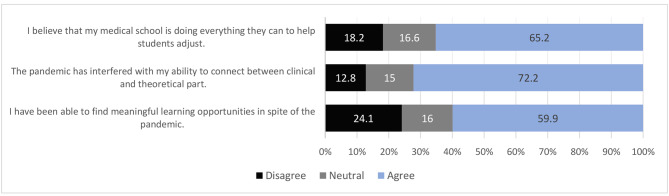
Evaluation of the effects on education

A total of 145 (77.5%) respiratory therapy students agreed that their clinical practice had been disrupted by the pandemic. Due to practical session cancellation, the number of respiratory therapy students who felt that they were less confident and prepared for the next level was 141 (75.4%). The complete outcomes of the questionnaire that are related to the effect on practice are illustrated in Fig. 1. Furthermore, most of the respondents’ ability to connect between clinical and theoretical part had been interfered because of the pandemic 135 (72.2%). Rest of the outcomes that are related the effect on education are illustrated in Fig. 2.

### Gender variable and the effects on practice and education

When analyzing the findings of association between the gender and the effects on practice and education, the findings revealed no significant association between female and male students and the disruption of the clinical practice, feeling less confident and prepared for the next level, and the interference of the pandemic in the ability to connect between clinical and theoretical part. On the other hand, Chi-squared test revealed a significant association between both gender and the desire to continue normal clinical rotation during this pandemic as female students had higher desire compared to male students (p value = 0.031). Regarding gender-based differences, female students had higher agreement response rate in regard to the received help from their school when compared to male response rate and statistical analysis revealed a significant difference (p value = 0.002). (Table 2).

**Table 2:** The association between the gender and the effects on practice and education (n = 187)

		Gender:			
		Male	Female	${X^2}$	p
		n (%)	n (%)		
My clinical practice has been significantly disrupted by the pandemic.				5.502	0.064
	Disagree	11 (37.9)	18 (62.1)		
	Neutral	10 (76.9)	3 (23.1)		
	Agree	70 (48.3)	75 (51.7)		
The pandemic has limited my ability to prepare competencies or skills I will need to start internship year / clinical rotation.				0.980	0.613
	Disagree	12 (41.4)	17 (58.6)		
	Neutral	10 (55.6)	8 (44.4)		
	Agree	69 (49.3)	71 (50.7)		
Covid-19 has shaped or influenced how I imagine spending my career.				0.228	0.892
	Disagree	12 (44.4)	15 (55.6)		
	Neutral	10 (50.0)	10 (50.0)		
	Agree	69 (49.3)	71 (50.7)		
I accept the risk that I maybe infected with covid-19 if I return to the clinical setting.				0.895	0.639
	Disagree	17 (45.9)	20 (54.1)		
	Neutral	9 (60.0)	6 (40.0)		
	Agree	65 (48.1)	70 (51.9)		
To the extent possible, respiratory therapist students should continue with normal clinical rotations during this pandemic.				6.961	# 0.031
	Disagree	12 (50.0)	12 (50.0)		
	Neutral	17 (73.9)	6 (26.1)		
	Agree	62 (44.3)	78 (55.7)		
RT students should be allowed to volunteer in summer training during this pandemic, even if there is no health care worker shortage.				1.137	0.566
	Disagree	9 (39.1)	14 (60.9)		
	Neutral	12 (46.2)	14 (53.8)		
	Agree	70 (50.7)	68 (49.3)		
In the event of healthcare worker shortage, RT students should be required to help clinically.				0.809	0.667
	Disagree	17 (45.9)	20 (54.1)		
	Neutral	10 (41.7)	14 (58.3)		
	Agree	64 (50.8)	62 (49.2)		
Due to practical session cancelation, my OSCE exam has been significantly affected.				0.131	0.937
	Disagree	16 (48.5)	17 (51.5)		
	Neutral	14 (51.9)	13 (48.1)		
	Agree	61 (48.0)	66 (52.0)		
Due to practical session cancelation, I feel less confident and less prepared for the next year/ next level.				0.143	0.931
	Disagree	16 (51.6)	15 (48.4)		
	Neutral	7 (46.7)	8 (53.3)		
	Agree	68 (48.2)	73 (51.8)		
I believe that my medical school is doing everything they can to help students adjust.				12.416	# 0.002
	Disagree	25 (73.5)	9 (26.5)		
	Neutral	17 (54.8)	14 (45.2)		
	Agree	49 (40.2)	73 (59.8)		
The pandemic has interfered with my ability to connect between clinical and theoretical part.				2.124	0.346
	Disagree	9 (37.5)	15 (62.5)		
	Neutral	12 (42.9)	16 (57.1)		
	Agree	70 (51.9)	65 (48.1)		
I have been able to find meaningful learning opportunities in spite of the pandemic.				0.344	0.842
	Disagree	22 (48.9)	23 (51.1)		
	Neutral	16 (53.3)	14 (46.7)		
	Agree	53 (47.3)	59 52.7)		

*Chi-squared test, n = number, # = p value < 0.05 consider significant

### University variable and the effects on practice and education

The analyzed results explained the association between the three universities and the effect on practice and education (Table 3). There was no significant association between the disruption of the clinical practice, the feeling of being less confident and prepared for the next level, and the interference of the pandemic in the ability to connect between clinical and theoretical part among the three universities. However, Chi-squared test revealed a significant association between university variable and students accepting the risk to be infected with COVID-19 if they return to the clinical setting (p value = 0.019). Comparing students at BMC to the other colleges, they were more opposed to get infected. Moreover, the university variable had a highly significant association with the students’ believing that their school did everything to help them in addition to a high significant association with the students’ ability to find meaningful learning opportunities during the pandemic (p value < 0.001) as determined by the test. Students at KAU were more likely not to receive the help from their school and were unable to find different learning resources to compensate for the lack of college’s offered resources compared to students from the other colleges.

**Table 3:** The association between the three universities and the effect on practice and education (n = 187)

		University				
		KSAUHS	KAU	BMC	${X^2}$	p
		n (%)	n (%)	n (%)		
My clinical practice has been significantly disrupted by the pandemic.					0.737	0.965**
	Disagree	10 (34.5)	10 (34.5)	9 (31.0)		
	Neutral	4 (30.8)	5 (38.5)	4 (30.8)		
	Agree	57 (39.3)	47 (32.4)	41 (28.3)		
The pandemic has limited my ability to prepare competencies or skills I will need to start internship year / clinical rotation.					3.969	0.410*
	Disagree	8 (27.6)	9 (31.0)	12 (41.4)		
	Neutral	6 (33.3)	8 (44.4)	4 (22.2)		
	Agree	57 (40.7)	45 (32.1)	38 (27.1)		
Covid-19 has shaped or influenced how I imagine spending my career.					0.477	0.976*
	Disagree	10 (37.0)	9 (33.3)	8 (29.6)		
	Neutral	9 (45.0)	6 (30.0)	5 (25.0)		
	Agree	52 (37.1)	47 (33.6)	41 (29.3)		
I accept the risk that I maybe infected with covid-19 if I return to the clinical setting.					11.731	# 0.019*
	Disagree	9 (24.3)	10 (27.0)	18 (48.6)		
	Neutral	9 (60.0)	3 (20.0)	3 (20.0)		
	Agree	53 (39.3)	49 (36.3)	33 (24.4)		
To the extent possible, respiratory therapist students should continue with normal clinical rotations during this pandemic.					3.821	0.431*
	Disagree	11 (45.8)	9 (37.5)	4 (16.7)		
	Neutral	11 (47.8)	5 (21.7)	7 (30.4)		
	Agree	49 (35.0)	48 (34.3)	43 (30.7)		
RT students should be allowed to volunteer in summer training during this pandemic, even if there is no health care worker shortage.					4.960	0.291*
	Disagree	9 (39.1)	8 (34.8)	6 (26.1)		
	Neutral	6 (23.1)	8 (30.8)	12 (46.2)		
	Agree	56 (40.6)	46 (33.3)	36 (26.1)		
In the event of healthcare worker shortage, RT students should be required to help clinically.					6.904	0.141*
	Disagree	15 (40.5)	16 (43.2)	6 (16.2)		
	Neutral	8 (33.3)	5 (20.8)	11 (45.8)		
	Agree	48 (38.1)	41 (32.5)	37 (29.4)		
Due to practical session cancelation, my OSCE exam has been significantly affected.					2.135	0.711*
	Disagree	13 (39.4)	12 (36.4)	8 (24.2)		
	Neutral	13 (48.1)	8 (29.6)	6 (22.2)		
	Agree	45 (35.4)	42 (33.1)	40 (31.5)		
Due to practical session cancelation, I feel less confident and less prepared for the next year/ next level.					1.667	0.797*
	Disagree	12 (38.7)	8 (25.8)	11 (35.5)		
	Neutral	6 (40.0)	6 (40.0)	3 (20.0)		
	Agree	53 (37.6)	48 (34.0)	40 (28.4)		
I believe that my medical school is doing everything they can to help students adjust.					19.324	# 0.001*
	Disagree	10 (29.4)	20 (58.8)	4 (11.8)		
	Neutral	18 (58.1)	4 (12.9)	9 (29.0)		
	Agree	43 (35.2)	38 (31.1)	41 (33.6)		
The pandemic has interfered with my ability to connect between clinical and theoretical part.					8.633	0.071*
	Disagree	10 (41.7)	6 (25.0)	8 (33.3)		
	Neutral	17 (60.7)	6 (21.4)	5 (17.9)		
	Agree	44 (32.6)	50 (37.0)	41 (30.4)		
I have been able to find meaningful learning opportunities in spite of the pandemic.					18.029	# 0.001*
	Disagree	14 (31.3)	24 (53.3)	7 (15.6)		
	Neutral	18 (60.0)	5 (16.7)	7 (23.3)		
	Agree	39 (34.8)	33 (29.5)	40 (35.7)		

*Chi-squared test, **Fisher-exact test, n = number, # = p value < 0.05 consider significant

### Academic Year Variable and the Effects on Practice and Education

The results described the association between the three academic years’ students and the effect on practice and education (Table 4). There was no significant association between the three academic years and the disruption of the clinical practice by the pandemic. On the other hand, Chi-squared test revealed a highly significant association between the students in the three academic years and the confidence and preparedness for the next academic year as more third year students felt less confident and less prepared compared to their seniors (p < 0.001). Furthermore, the same pattern was observed for the association between the students in the three academic years and the interference of the pandemic in the ability to connect between clinical and theoretical part as determined by the test (p < 0.001). In addition, the test revealed a significant association between the academic year and students’ desire to volunteer in summer training even without the Health Care Worker (HCW) shortage (p value = 0.046). Similar to the other associations, third year students were more desired to volunteer in summer training when compared to their fifth year students. Moreover, the test showed that academic year variable had a significant association with the students’ believing that their medical school did everything to help them (p = 0.033). Among the three academic years, third year students were more likely to receive less help from their school.

**Table 4:** The association between the three academic years’ students and the effect on practice and education (n = 187)

		Academic Year:				
		Third	Fourth	Fifth (Intern)	${X^2}$	p
		n (%)	n (%)	n (%)		
My clinical practice has been significantly disrupted by the pandemic.					0.876	0.928
	Disagree	12 (41.4)	9 (31.0)	8 (27.6)		
	Neutral	5 (38.5)	4 (30.8)	4 (30.8)		
	Agree	58 (40.0)	54 (37.2)	33 (22.8)		
The pandemic has limited my ability to prepare competencies or skills I will need to start internship year / clinical rotation.					8.282	0.082
	Disagree	6 (20.7)	11 (37.9)	12 (41.4)		
	Neutral	8 (44.4)	5 (27.8)	5 (27.8)		
	Agree	61 (43.6)	51 (36.4)	28 (20.0)		
Covid-19 has shaped or influenced how I imagine spending my career.					5.020	0.285
	Disagree	9 (33.3)	10 (37.0)	8 (29.6)		
	Neutral	11 (55.0)	3 (15.0)	6 (30.0)		
	Agree	55 (39.3)	54 (38.6)	31 (22.1)		
I accept the risk that I maybe infected with covid-19 if I return to the clinical setting.					4.160	0.385
	Disagree	15 (40.5)	16 (43.2)	6 (16.2)		
	Neutral	6 (40.0)	3 (20.0)	6 (40.0)		
	Agree	54 (40.0)	48 (35.6)	33 (24.4)		
To the extent possible, respiratory therapist students should continue with normal clinical rotations during this pandemic.					3.696	0.449
	Disagree	7 (29.2)	10 (41.7)	7 (29.2)		
	Neutral	7 (30.4)	8 (34.8)	8 (34.8)		
	Agree	61 (43.6)	49 (35.0)	30 (21.4)		
RT students should be allowed to volunteer in summer training during this pandemic, even if there is no health care worker shortage.					9.670	# 0.046
	Disagree	7 (30.4)	10 (43.5)	6 (26.1)		
	Neutral	7 (26.9)	7 (26.9)	12 (46.2)		
	Agree	61 (44.2)	50 (36.2)	27 (19.6)		
In the event of healthcare worker shortage, RT students should be required to help clinically.					4.009	0.405
	Disagree	14 (37.8)	11 (29.7)	12 (32.4)		
	Neutral	9 (37.5)	7 (29.2)	8 (33.3)		
	Agree	52 (41.3)	49 (38.9)	25 (19.8)		
Due to practical session cancelation, my OSCE exam has been significantly affected.					4.759	0.313
	Disagree	12 (36.4)	10 (30.3)	11 (33.3)		
	Neutral	11 (40.7)	7 (25.9)	9 (33.3)		
	Agree	52 (40.9)	50 (39.4)	25 (19.7)		
Due to practical session cancelation, I feel less confident and less prepared for the next year/ next level.					23.622	# <0.001
	Disagree	4 (12.9)	12 (38.7)	15 (48.4)		
	Neutral	6 (40.0)	2 (13.3)	7 (46.7)		
	Agree	65 (46.1)	53 (37.6)	23 (16.3)		
I believe that my medical school is doing everything they can to help students adjust.					10.500	# 0.033
	Disagree	21 (61.8)	10 (29.4)	3 (8.8)		
	Neutral	10 (32.3)	14 (45.2)	7 (22.6)		
	Agree	44 (36.1)	43 (35.2)	35 (28.7)		
The pandemic has interfered with my ability to connect between clinical and theoretical part.					20.679	# <0.001
	Disagree	3 (12.5)	10 (41.7)	11 (45.8)		
	Neutral	11 (39.3)	5 (17.9)	12 (42.9)		
	Agree	61 (45.2)	52 (38.5)	22 (16.3)		
I have been able to find meaningful learning opportunities in spite of the pandemic.					1.762	0.78
	Disagree	17 (37.8)	19 (42.2)	9 (20.0)		
	Neutral	13 (43.3)	11 (36.7)	6 (20.0)		
	Agree	45 (40.2)	37 (33.0)	30 (26.8)		

*Chi-squared test, n = number, # = p value < 0.05 consider signifi

## Discussion

COVID-19 pandemic significantly affected the clinical practice of many healthcare fields, especially allied health students [9–12]. Around 54% of ophthalmology trainees from several countries had reported that their training was severely impacted by the pandemic [15]. This was supported by another study reported that restrictions imposed during the COVID-19 pandemic had negatively affected medical student clinical practice as it showed reduction in essential clinical skills required for medical students [12]. In this study, 77.5% of RT students reported that they had major disturbances in their clinical training. This might be due to the fact that most of COVID-19 patients where admitted to ICU and entrance restrictions were applied to all patients relatives as well as training students. However, the disturbance was not significantly associated with any of the variables, which might be generalized to all students and did not affect a specific group. Moreover, assessing the long-term effect, including two and three years following the pandemic is recommended.

Students have accepted the risk of being infected with COVID-19 if they return to clinical settings. Previous studies showed that around 83% of United State (US) medical students agreed to the risk of being infected [14]. Moreover, a study conducted in Duke-NUS Medical School in the Republic of Singapore revealed that around 64% of the students have accepted the risk of being infected [16]. According to our study, 72.2% of RT students had the desire to continue clinical training and agreed to accept the risk of being infected. RT female students had higher desire compared to male students. These desires could be due to the gender-based variability, sense of professional responsibility, and a self-motivation to take the chance to improve clinical capacity. On the other hand, comparing students at BMC to the other colleges, they were more opposed to get infected. This factor needs to be further investigated as some demographic data might have an impact of this association.

Students have agreed to volunteer in clinical settings during summer training even without HCW shortage. In the US, around 63% of medical students had the desire to volunteer [14]. On the other hand, RT students were more likely to volunteer with agreement percentage of 73.8%. Third year RT students were more desired to volunteer in summer training when compared to their fifth-year students. This might suggest that third year students thought of volunteering as an alternative plan to compensate for the cancelled clinical rotations.

COVID-19 pandemic had disrupted medical students’ confidence. In the US, around 50% of medical students felt less confident and less prepared for the next level [14]. Another study has supported that over 50% of medical students showed decreased confidence in clinical settings and feel unprepared clinically due to the pandemic [17]. In this study, statical analysis revealed that the percentage of respiratory therapy students who felt less confident and prepared for next academic year was 75.4%, which considered a high percentage when compared to medical students in other studies. Majority of third year RT students felt less confident and less prepared compared to their senior. Third year is the foundation year for practical sessions, so the association might be due to students’ concerns about their performance in the next academic years.

COVID-19 pandemic affected educational systems globally and colleges may or may not have helped students accommodate changes occurred during the academic year. The received help to RT students have varied among gender, universities, and academic year. Regarding gender-based differences, male students had higher disagreement response rate in regard to the received help from their school when compared to female response rate. Regarding university-based differences, students at KAU were more likely not to receive the help from their school. Among the three academic years, third year students were less likely to receive help from their school. These variations may be due to miscommunication. Female students, other universities, and other academic years might have communicated with their schools in regards of the obstacles they faced during the pandemic, which may lead to increase the chances of schools response to their inquiries and provide them the help they need. Previously published study about US medical students have reported that around 72% of participants received the help they need which was achieved by daily communication updates [14]. This shows that communication was one of the factors that affected students’ virtual education.

During the pandemic, different meaningful learning experiences, such as virtual online courses, self-guided study electives, COVID-19 related volunteering opportunities, and more were available for students to capture and compensate for the lack of college’s offered resources. Nearly 72% of medical students in US reported that they were able to find these experiences [14]. Furthermore, up to 92% of medical students combined their coursework with other learning opportunities [17]. On the other hand, 59.9% of RT students were able to find resources compared to other studies. KAU students were less likely to capture these experiences compared to other colleges. Given that some demographic information could affect this association, this component needs to be further investigated.

The findings of this study that pertained to the effect of the pandemic on respiratory therapy students was consistent with the findings from previous studies [12, 14–17]. However, in our study, students were asked if the pandemic had interfered with their ability to connect between clinical and theoretical part, around 72.2% responded positively. The lack of connection between clinical and theoretical part was significantly associated with the academic year. The pandemic had interfered more likely with third year students, which might be due to the theoretical content of their curriculum. It includes the basic skills that need to be practiced, and without practicing these skills, it might be easily forgotten for most of the students. In addition, virtual education with lack of in-person training might be inadequate for gaining the basics competencies required for RT, such as assessment and physical examination skills. The lack of connection between clinical and theoretical part is a new challenge that this study has identified, which needs to be investigated on a larger scale to address a potential solutions.

### Strengths

To the best of our knowledge, there is a lack of research in the field of COVID-19 effects on healthcare professions, especially respiratory therapy. Moreover, the importance of this study is to investigate the direct impact of COVID-19 virtual training on the quality of respiratory therapy clinical years. This study is considered as one of the first studies to shed the light on the effect of the pandemic that has interfered with RT students’ ability to connect between clinical and theoretical part.

### Limitations

Regarding the study design (cross-sectional), this study assessed the current effect of COVID-19 on respiratory therapy students; however, there was no follow-up period for the respondents. As a result, the long-term impact of the pandemic on the transition period over the study years was not yet determined in this study. Another limitation of our study was the questionnaire response rate of fifth year students. It was challenging to reach fifth year students and collect their response as they were in their internship year. To increase response rates, we attempted to send the questionnaire directly through fifth year respective class representatives.

### Recommendations

Future research is guaranteed to investigate the long-term impact on students’ performance in the clinical practice after graduation. Another recommendation for future research is to investigate the useful colleges’ preparations during the pandemic that helped students in their education and practice. Moreover, it is important to investigate the different learning resources that were found by students during the pandemic to compensate for the lack of college’s offered resources.

## Conclusion

The responses from the three universities similarly recognized a disruption to the respiratory therapy students’ clinical practice, education, and confidence. The majority of students have reported that the pandemic had interfered with their practice, which affected not only their ability to connect the clinical and theoretical part but also their confidence about their performance and preparedness in the next years. Urgent alternative solutions ought to be adopted by medical schools to deliver a comprehensive medical education and effective practical sessions for respiratory therapy students that do not require the physical attendance. This may enable students to practice the learned skills during clinical years and prevent major disruption and delay to their clinical and theoretical education and their ability to connect both parts. Furthermore, respiratory therapy students should be allowed to volunteer clinically during the pandemic so that they provide help and get benefit for their training. This might help the students to gain confidence and be prepared for the next phase.

## Data Availability

The datasets used and/or analyzed during the current study are available from the corresponding author on reasonable request.

## List of abbreviations

RT: Respiratory Therapist – Respiratory Therapy
COVID-19: The Novel Coronavirus Disease 2019
ICU: Intensive Care Unit
KSAUHS: King Saud bin Abdulaziz University for Health Sciences
KAU: King Abdulaziz University
BMC: Batterjee Medical College
SPSS: Statistical Package for the Social Sciences
HCW: Health Care Worker
OSCE: Objective Structured Clinical Examination
US: United States
